# Comparison of Upper Limb Translocated Femoral Vein and Prosthetic Arteriovenous Bridge Grafts

**DOI:** 10.7759/cureus.6219

**Published:** 2019-11-22

**Authors:** Muhammad Asad Moosa, Fareed A Shaikh, Madeeha Ali, Abdus Salam, Ziad Sophie, Nadeem Siddiqui

**Affiliations:** 1 Surgery, Aga Khan University Hospital, Karachi, PAK; 2 General Surgery, Aga Khan University Hospital, Karachi, PAK; 3 Surgery, The Aga Khan University, Karachi, PAK

**Keywords:** dialysis access, femoral vein, avbg, vein translocation, arteriovenous fistula

## Abstract

Introduction

Native veins are an ideal option for dialysis in a patient with chronic kidney disease (CKD) as compared to a prosthetic graft. Femoral vein (FV) translocation to the upper arm is also an alternative to a prosthetic graft as reported in the literature when all options of using the native veins of the arms are exhausted. Thus, we aimed to compare the patency of the upper limb translocated FV arteriovenous fistula (AVF) with a prosthetic arteriovenous bridge graft (AVBG).

Methods

It is a retrospective cohort study that was conducted in the Department of Vascular Surgery, Aga Khan University Hospital. It included adult patients who underwent either upper arm translocation of FV or prosthetic AVBG using the consecutive purposive sampling technique. There were a total of 10 patients who underwent FV translocation AVF and 20 patients who had AVBG in the upper arms.

Results

A total of 30 patients were included in the study. Of these 30 patients, 10 underwent FV translocation AVF and the remaining 20 had AVBG. There was a significant difference in the mean operating time of the two surgeries. The mean operating time in FV translocation was 223 (± 41.5) minutes and in those with AVBG, the mean operating time was 100 (±26.5) (p= <0.001). There was no significant difference in the total length of hospital stay in both procedures performed. The primary patency rate for FV translocation was 90% and 95% in AVBG (p=1.00). Ten percent of FV translocation had a primary failure rate compared with that of AVBG, which was 5% (p=1.00). The mean follow-up period was 61 weeks in the FV translocation group and 64 weeks in the AVG group.

Conclusion

There was no significant difference in both groups in terms of patency, length of hospital stay, and fewer complications were observed in the FV translocation group as compared with the AVBG group.

## Introduction

The goal of long-term vascular access is rapid and repeated exchange of blood to the body’s circulation with fewer complications [1]. Establishing a native vein arteriovenous fistula is always not possible, especially in patients with old age and diabetes [2-3]. In such circumstances, vascular access grafts are used [4]. However, prosthetics are associated with lower survival, higher complication rates, and limited graft patency due to repeated puncturing [5-6]. The alternate is the femoral vein (FV), which has the added benefit of a larger diameter (6-10 mm) and had been used in situations where upper limb veins were exhausted or in repeated prosthetic graft failures [7-11]. However, FV translocation is also associated with added complications, including steal syndrome, wound complications, and venous leg swellings [6]. To the best of our knowledge, there is no published literature comparing FV translocation to the upper limb with an arteriovenous bridge graft (AVBG) in the upper limb. Therefore, we conducted a retrospective comparative study with the primary objective to compare the patency of FV translocation AVF and AVBG in the upper limb. Our secondary objectives were to compare the overall wound-related complications, duration of surgery, and length of hospital stay.

## Materials and methods

This is a retrospective cohort study that was conducted at the Department of Surgery, Aga Khan University Hospital, Karachi, Pakistan. Approval from the university’s ethical review committee was obtained (4581-Sur-ERC-16). The duration of this study was from January 1, 2015, to February 28, 2018. FV translocation was performed in patients with exhausted veins and who were young, non-obese, and able to withstand two different wounds and prolonged surgery. As compared to this, AVBG was on priority for patients who were elderly, frail, and with multiple comorbidities. So during the study period, adult patients who underwent either upper arm translocation femoral vein grafts (Group A) or prosthetic arteriovenous bridge grafts (AVBG) (Group B) for permanent hemodialysis access were included.

In the AVBG group, a 6-millimeter polytetrafluoroethylene (PTFE) graft was used in every procedure and the graft was tunneled subcutaneously in the arm after two separate incisions in the axilla and lower arm. The graft was anastomosed in an end-to-side anastomosis fashion with the brachial artery and the axillary vein. In the FV translocation group, a longitudinal incision in the thigh, extending from the groin to the lower thigh, was given. The femoral vein was dissected along its course up till the knee and harvested. Two separate incisions were given in the arm (one in the axilla and one in the lower arm). The harvested vein was tunneled subcutaneously and anastomosed in an end-to-side fashion with the brachial artery and the axillary vein.

One-year follow-up was gained through a phone call interview, and relevant questions regarding fistula patency and other complications were asked. If a follow-up in the clinic was required, the patients were asked to visit the clinic free of cost. Patients with incomplete follow-up (less than one year), patients with incomplete medical records, and patients who expired or were not available at the time of the phone interview were excluded. The sampling technique was consecutive purposive (patients were recruited who fulfilled the criteria for inclusion until the required sample was achieved). During the specified study period, 10 patients underwent FV translocation AVF. To compare this, 20 patients who underwent AVBG during the same time period and of the same age range were enrolled in the study. Data were collected by means of a questionnaire that was filled for the sample population by the data analyst who was a member of the team. All the data were collected retrospectively from the patients’ medical records.

Data regarding the patients' demographics, type of procedure, complications, including wound dehiscence, hematoma, surgical site infection, duration of surgery, and length of hospital stay, were collected from the patient’s medical records. Data were analyzed using Statistical Package for Social Sciences (SPSS) version 21.0 (IBM Corp., Armonk, NY) [12]. Mean ± standard deviation was computed for numerical variables. T-test, chi-square test, and correlation analysis were used for variable comparison within the two groups. Statistically, the level of significance was taken at p<0.05.

## Results

A total of 10 patients underwent FV translocation and 20 patients were selected from the AVBG group. The median age for patients receiving FV translocation was 47.5 (IQR 19-63) years and for those with AVBG, it was 55 (IQR 22-68) years. The major comorbidities in these patients were diabetes (60% in FV vs 75% in AVBG) and hypertension (70% in FV and 95% in AVBG) (Table 1).

**Table 1:** Details of patients of the two groups (FV v/s AVBG) FV: femoral vein; AVBG: arteriovenous bridge graft

	FV (n=10)	AVBG (n=20)	p-value
Median Age (IQR )	47.5 (19-63)	55 (22-68)	
Diabetes Mellitus (DM)	6 (60%)	14 (75%)	
Hypertension (HTN)	7 (70%)	18 (95%)	
Previous Access Surgeries (Mean +/- SD)	2 (±1.3)	1 (±0.94)	
Operating Time in Mins (Mean +/- SD)	223 (±41.5)	100 (±26.5)	<0.001
Length of Stay (Mean +/- SD)	3 (±0.568)	2 (±0.394)	0.154
Mean Follow-up (Weeks)	61.40 (± 6)	63.95 (± 8)	0.38

There was a significant difference in the mean operating time of the two surgeries. The mean operating time for an FV translocation was 223 (± 41.5) minutes and for those with an AVBG, the mean operating time was 100 (± 26.5) (p<0.001). There was no significant difference in the total length of hospital stay in both groups of patients. Half of the FV translocations (n=5) were matured and used before six weeks (Table 2). However, 80% of the AVBG (n=16) were used and matured before six weeks.

**Table 2:** Maturation time AVBG: arteriovenous bridge graft

Time of First Use	FV (n=10)	AVBG (n=20)	p-value
Less than 6 weeks	5 (50%)	16 (80%)	0.115
At 6 weeks	5 (50%)	4 (20%)	0.115

In terms of patency, the primary patency rate for FV translocation was 90%, and it was 95% in AVBG (p=1.00). Ten percent of FV translocation had a primary failure rate as compared with that of AVBG, which was 5% (p=1.00) (Table 3).

**Table 3:** Patency of the two groups (FV v/s AVBG) FV: femoral vein; AVBG: arteriovenous bridge graft

	FV (n=10)	AVBG (n=20)	p-value
Patency	9 (90%)	19 (95%)	1.000
Primary Failure	1 (10%)	1 (5%)	1.000

Thigh wound infection was observed in only one patient with FV translocation. An ischemic complication, including steal syndrome, was seen in three patients with AVBG and not seen in the FV translocation group (Table 4). One patient with FV translocation required an additional procedure to assist patency but the number increased in patients with AVBG, i.e. a total of three patients. Graft infection and central venous stenosis were seen in one patient with AVBG (Table 4).

**Table 4:** Complications in both groups FV: femoral vein; AVBG: arteriovenous bridge graft

	FV (n=10)	AVBG (n=20)
Wound Infection	1	-
Postop Hematoma	-	-
Hand Ischemia/Steal	-	3
Intervention to Assist Patency	1	3
Graft Infection	-	1
Central Vein Stenosis	-	1

## Discussion

Femoral vein translocation was first described in a case report in 2000 by Huber et al. [13] and then later in an outcome study in 2004 [7]. After that, very few published studies have discussed the pros and cons of this technique. FV translocations are more invasive and a time-consuming procedure when compared to standard AVBG and careful patient selection is very important. It also gives the advantage of being a native vein fistula with less infectious complications with comparable patency. It is advisable that it should be offered to a younger population who can withstand a long surgical procedure without excessive morbidity. The primary aim of this study was to perform a comparison analysis of the two techniques in a dialysis vascular access creation. This comparison will later help in selecting appropriate dialysis access for a patient. Another reason for this comparison is to cut down on the cost of the prosthetic graft used in AVBG if possible, which is an added treatment cost. Maintaining the treatment cost for a dialysis patient in our society is very crucial, as almost all the patients are self-payers.

The maturity time in our study of FV translocations was five weeks, which, in comparison to a case series from Huber et al., showed a maturity time of seven weeks [7]. Another study showed similar results for fistula maturation, which was four weeks [13]. The primary patency rate of FV translocation in our study was 90% in the FV, which is comparable to the parent study done by Huber et al. who had a patency rate of 96% in FV translocation [14]. Overall, the translocation of FV was associated with fewer complications in our study. But, Huber et al. reported the breakdown of the vein harvest wound as a known complication of an FV translocation [7]. However, the complication rate from our center was less, and only 10% of our patients had a wound complication. Another study from Rueda, et al. showed comparable results in length of stay, but slightly higher complication rates, which were 45% [10]. Wound complications were higher in their study because they did not use a drain in their initial patients, this was then decreased to 13% when initiating the use of drains [10]. Drains were often used in our FV translocation patients with thigh wounds.

Interestingly, ischemic complications were higher in the AVBG group when compared to the FV translocation group, which is an important finding in our study. Higher ischemic complications may be explained by the presence of peripheral arterial disease in AVBG patients. Ischemic complications in both techniques have been reported previously by Matsuura, et al. [15] who showed 3% of hand ischemia in AVBG and 2% in the femoral vein graft. However, this can be avoided by getting preoperative imaging of the arteries, particularly in elderly patients, which was not performed. This assessment is not a routine in our setting due to financial constraints.

Selecting an appropriate dialysis access option in patients who have all the upper limb veins exhausted is a critical step. The results of this study favor FV translocation in younger patients with a longer life expectancy on dialysis and having a low risk of postoperative morbidities. The major limitations of this study is the low number of patients in the FV translocation group, unequal sample size between the two groups for comparison, and due to its retrospective nature, an inability to get complete information because of the inadequate documentation on follow-ups. We plan to further modify the results of this study by collecting larger amounts of data and comparing them in a prospective manner and with at least two years of follow-up post-intervention. The major strengths that this study provides centers on the first comparative study to see the difference in one-year patency between the two techniques.

## Conclusions

There was no difference in terms of patency between the two procedures. However, the surgical time necessary to perform an FV translocation procedure was higher than for AVBG. No difference was noted in the length of hospital stay, and postoperative complications were higher in the AVBG group. Upper limb translocation of the FV can be an alternative option in patients with exhausted upper limb veins, as the patency rate for an upper limb translocated FV is exceptional and comparable to AVBG. However, knowing the magnitude of morbidities, the patient population for such a procedure should be selected after complete and careful assessment.
